# HIV STIGMA AND COGNITION AMONG OLDER WOMEN LIVING WITH HIV

**DOI:** 10.1093/geroni/igae098.4206

**Published:** 2024-12-31

**Authors:** Thi Vu, Jenni Wise, Deborah Jones Weiss, Monica Diaz, Aruna Chandran, Sheri Weiser, Leah Rubin, Joan Monin

**Affiliations:** Yale University School of Public Health, Charlotte, North Carolina, United States; University of Alabama at Birmingham, Birmingham, Alabama, United States; University of Miami Miller School of Medicine, Miami, Florida, United States; School of Medicine, University of North Carolina at Chapel Hill, Chapel Hill, North Carolina, United States; The Bloomberg School of Public Health, Johns Hopkins University, Baltimore, Maryland, United States; University of California San Francisco, San Francisco, California, United States; The Bloomberg School of Public Health, Johns Hopkins University, Baltimore, Maryland, United States; Yale School of Public Health, New Haven, Connecticut, United States

## Abstract

Internalized HIV stigma refers to the negative beliefs, feelings, and attitudes that people living with HIV (PLHIV) adopt about themselves due to societal HIV stigma. Internalized HIV stigma negatively impacts mental health. Up to 55% of PLWH experience HIV-associated neurocognitive disorders. This study examines longitudinal associations between internalized HIV stigma and cognition among women aged 50+ living with HIV in the historic Women’s Interagency HIV Study. Internalized HIV stigma was measured between 2013-2015 using the negative self-image sub-scale of the HIV stigma scale. Seven cognitive domains (executive function, processing speed, attention/working memory, verbal learning, verbal memory, verbal fluency, and fine motor function) were assessed using a validated cognitive battery at baseline (2013-2017) and 2-3 years later. Demographically adjusted T-scores were calculated for each domain with higher scores indicating better performance. A global cognition score was computed by averaging the domain-specific scores. Linear regression models adjusted for age, race, ethnicity, cognition at baseline, average annual income, undetectable viral load, smoking history, recent non-prescription drug use, menopausal status, depression, and alcohol use. Participants’ (N=760) mean age was 54 years; 61% identified as Black/African American; 13% were Hispanic; and 54% had an annual income below $12,000. The mean HIV stigma score was 1.78 (SD=0.64). Higher internalized HIV stigma was associated with poorer global cognitive function (B= -0.68, p< 0.05), verbal learning (B= -1.42, p< 0.05), and verbal memory (B= -1.40 p< 0.05) at time 2. Findings suggest assessing and monitoring HIV stigma could have beneficial cognitive effects for older women aging with HIV.

